# Designing for Patient-Centered Care and Equity in Virtual Hospital-at-Home Models: Quality Improvement Initiative Using Experience-Based Co-Design

**DOI:** 10.2196/79679

**Published:** 2025-11-05

**Authors:** Mahabhir Kandola, Emma Wong, Robert Paquin, Kamal Arora, Harroop Sharda, Roman Deol, Mary Jung, Maria Montenegro, Megan MacPherson

**Affiliations:** 1 Department of Virtual Health and Department of EDI and Language Services, Fraser Health, 13450 102 Ave, Surrey, V3T0H1, Canada, 1 6045616605; 2 King's College London, 5-11 Lavington StreetLondon, United Kingdom; 3 British Columbia Institute of Technology, Burnaby, BC, Canada; 4 University of British Columbia, Kelowna, BC, Canada; 5 University of British Columbia, Vancouver, BC, Canada

**Keywords:** community-based participatory research, telemedicine, health equity, South Asian people, artificial intelligence, AI

## Abstract

**Background:**

The rapid expansion of virtual care during COVID-19 accelerated the development of virtual hospital-at-home models, which deliver hospital-level care in patients’ homes through remote monitoring, virtual communication, and in-person support when required. While the virtual hospital-at-home model offers the potential to improve patient-centered care and health equity, rapid implementation often overlooks culturally diverse and underserved populations, including South Asian communities who experience disproportionate chronic disease burden and barriers to accessing culturally relevant services. Strategies are needed to ensure equitable design and adoption of virtual hospital-at-home models.

**Objective:**

This study used an experience-based co-design (EBCD) approach to engage patients, caregivers, clinicians, and community organizations in shaping a regional virtual hospital-at-home strategy within the Fraser Health Authority, British Columbia, Canada. The aim was to identify barriers, facilitators, and equity-focused solutions to inform future implementation.

**Methods:**

We conducted a five-stage EBCD quality improvement process in the Fraser Health Authority, British Columbia, including (1) forming a multidisciplinary steering committee, (2) reviewing health care provider experiences, (3) interviewing South Asian patients and caregivers, (4) hosting a co-design workshop to develop solutions, and (5) sharing back findings.

**Results:**

Participants identified barriers, including digital literacy, language, and trust in virtual care. The co-designed solutions focused on culturally tailored education, hybrid digital training, caregiver inclusion, and community-driven engagement strategies.

**Conclusions:**

EBCD enabled the development of inclusive and actionable strategies to improve virtual hospital-at-home services. The findings highlight the importance of ongoing community collaboration to ensure equity in virtual care innovation.

## Introduction

### Background

The rapid expansion of virtual care delivery in response to COVID-19 has paved the way for the development of virtual hospital-at-home models, which are models of care that mirror the structure and functions of traditional inpatient wards, but enable eligible patients to receive hospital-level care in their own homes through a combination of remote monitoring, virtual communication, and in-person support when needed [1]. Unlike general telemedicine or outpatient virtual visits, which typically provide episodic or consultative care, the virtual hospital-at-home model aims to deliver a continuous and integrated alternative to acute hospital care.

Virtual hospital-at-home services hold promise in improving patient-centered care and enhancing health equity within communities [2]; however, the rapid implementation of virtual care during COVID-19 lacks comprehensive patient-centered development and evaluation, potentially marginalizing already underserved populations (eg, older adults, people with disabilities, and culturally diverse communities) [3]. Without effective strategies for continuous improvement, virtual care risks perpetuating inequitable and low-quality care, undermining the very goals they aim to achieve [4]. In Canada, South Asian communities represent a significant proportion of residents who have historically been underserved and experience substantial barriers related to language, cultural relevance, and access to health services [5]. South Asians in Canada have higher rates of chronic diseases compared to other racialized groups, including cardiovascular disease, diabetes, and hypertension [6,7]—partly due to social determinants of health (eg, access to education, employment opportunities, food security, experiences of discrimination, and access to culturally appropriate care) [8-10]. Given the inequities and disparities faced by South Asian community members within Canada, innovative care strategies are needed to enhance access to culturally relevant care and improve health outcomes.

### Challenges

Key virtual care challenges include the following: (1) the digital divide: disparities in technology access and digital literacy disproportionately exclude vulnerable populations, such as older adults and culturally diverse communities, from the benefits of virtual care [11,12]; (2) workforce strain: clinicians face challenges navigating new technologies and workflows and often lack the time to learn new systems and technologies contributing to burnout and hindering virtual care adoption [13]; (3) rapid technological evolution: virtual health requires continuous innovation and adaptability to ensure sustainability, interoperability, and alignment with patient needs [14,15]. In this paper, we specifically address challenges related to equitable access and patient-centered engagement in virtual hospital-at-home services.

### Fraser Health Setting

These challenges are particularly salient within the Fraser Health Authority, the largest regional health authority in British Columbia, Canada, which provides care to more than 2 million residents across 20 diverse communities [16]. Fraser Health serves 75% of the South Asian residents in the province, and 1 in 5 Fraser Health residents identifies as South Asian [17]. Considering Fraser Health’s diverse population and geographical spread, the virtual hospital-at-home model has the potential to improve care quality and access while alleviating pressure on hospital resources. Fraser Health is designing a regional program and strategic implementation guidelines for a virtual hospital-at-home service with five primary objectives: (1) increase overall system capacity, (2) improve patient flow and efficiency, (3) reduce hospital-acquired harm, (4) enhance patient and essential care partner experience, and (5) enhance clinician experience.

At the time of this study, the only active unit was the virtual psychiatric unit (VPU), launched in fall 2022 [18]. The VPU virtually provides hospital-level psychiatric care, but the long-term vision for Fraser Health’s virtual hospital-at-home strategy is to expand to other clinical areas (not yet specified). This project did not evaluate the VPU directly, but instead sought to engage community members in shaping the design of future virtual hospital-at-home models across a range of clinical areas.

### This Project

This study uses a design thinking approach, experience-based co-design (EBCD) [19], to help inform the development of the Fraser Health virtual hospital-at-home service. EBCD is a participatory, user-centered approach to quality improvement that engages those closest to the area for improvement. In this project, patients, caregivers, clinicians, and decision-makers were included to explore areas for improvement and to co-design service improvements based on lived experiences. EBCD is based on the premise that co-design can lead to experience-focused health care services, which can result in improved outcomes in clinical effectiveness and safety [20]. This multistage service improvement approach captures the experiences and emotions of service users by understanding key events or “touchpoints” during their service journey [20].

Importantly, EBCD has been recognized as a vehicle for patient empowerment and equity, shifting traditional power structures by positioning patients as co-producers in health care improvement [21]. In public health, more broadly, co-creation models such as EBCD have been intentionally used to address health inequities by involving marginalized communities across all phases of intervention development, from agenda-setting to evaluation [22]. In addition, EBCD has been shown to help flatten epistemic inequities by equally valuing all users’ experiences and dismantling entrenched power imbalances in co-design processes [23]. By centering on the lived experiences of patients and caregivers, particularly from underserved communities, EBCD ensures that service improvements prioritize equity alongside quality and safety.

## Methods

### Overview

EBCD is a quality improvement methodology that is typically structured into 6 stages [19]. This study adapted the traditional 6-stage EBCD framework into 5 stages, as the final celebratory event was integrated into steering committee debrief sessions rather than conducted as a stand-alone event. A core component of EBCD is the creation of a short film based on interviews with participants, which is used to spark discussions between those providing and receiving care at a co-design event. This study consisted of 5 stages, and this paper describes the process of using EBCD to help inform the Fraser Health virtual hospital-at-home service (Table 1).

**Table 1.** Stages of the experience-based co-design approach.

Stage	Key activities	Participants	Key outputs
Stage 1: setting up and steering committee	Established steering committee and monthly meetings	PICS^a^ society, SAHI^b^, Virtual Health, EDI^c^ team, and researchers	Guidance on study approach and culturally relevant methods
Stage 2: observing service delivery and staff experiences	Interviews with health care providers	7 staff (n=5, 71% physicians and n=2, 29% nurses)	Insights on barriers and opportunities for virtual hospital-at-home models
Stage 3: gathering patient experiences and touchpoint film	Interviews with South Asian community members and film creation	20 patients and caregivers (9 in film)	Short touchpoint film highlighting key experiences
Stage 4: co-design event	Workshop using film to guide problem-solving	Patients, caregivers, clinicians, and steering committee	Solutions for 5 key themes: patient-centered care, technology, training, community engagement, and process improvement
Stage 5: post–co-design analysis and uptake	Thematic analysis and follow-up meetings	Steering committee and the virtual hospital development team	Recommendations shared, implementation planning, and reflections

a PICS: Progressive Intercultural Community Services.

b SAHI: South Asian Health Institute.

c EDI: Equity, Diversity, and Inclusion.

### Stage 1: Setting Up and Establishing a Steering Committee

Members from the Progressive Intercultural Community Services (PICS) society, Fraser Health’s Virtual Health department, Fraser Health’s South Asian Health Institute, Fraser Health’s Equity, Diversity, and Inclusion team, and researchers were invited to join the steering committee. These groups were chosen for their understanding of the underserved communities (particularly South Asian communities), their roles in the health care system, and their experience with virtual care services and community-based research methods, ensuring a comprehensive perspective for the project. The committee met monthly to discuss project updates, provide feedback on the research approach, and assist in refining the study’s methods.

### Stage 2: Observing Service Delivery and Gathering Staff Experiences

To contextualize the development of virtual hospital-at-home services within Fraser Health, we incorporated findings from a previous qualitative study with 7 health care providers (n=5, 71% physicians and n=2, 29% registered nurses) regarding their experiences with existing remote patient monitoring services. We conducted approximately 30-minute, semistructured one-on-one interviews with health care providers across Fraser Health. The semistructured interview guide was developed in consultation with the Virtual Health department to ensure that the questions were relevant, actionable, and aligned with the information needs of the remote patient monitoring program. Interviews explored health care providers’ experiences referring patients to remote patient monitoring programs, barriers encountered, perceived advantages and disadvantages of referral, and suggestions for program improvements. These interviews were included to inform this study, as remote patient monitoring is an integral aspect of virtual hospital-at-home models. Remote patient monitoring allows clinicians to track patient status in real time and supports proactive care delivery. For full details on the methods used, refer to the study by N Moghaddam, M Montenegro, and M MacPherson (unpublished data, 2025).

### Stage 3: Gathering Patient Experiences and Creating Touchpoint Film

#### Overview

Qualitative interviews were conducted with 20 South Asian community members in multiple languages to better understand the attitudes, experiences, and perceptions of patients and caregivers toward virtual hospital-at-home models. South Asian communities are among the largest racialized groups within the Fraser Health region. These interviews identified perceived barriers and preferences regarding virtual hospital-at-home models. Moreover, 30- to 60-minute, one-on-one semistructured interviews were conducted virtually via Microsoft Teams. Technical support was available through the PICS society to ensure participant access. The topic guide was adapted from previous Fraser Health Virtual Health workshops, revised with feedback from South Asian community members, and reviewed by the plain language team for clarity. The guide covered perceptions of and attitudes toward the virtual hospital-at-home model, including perceived benefits, concerns, and strategies for community engagement. During the consent process, participants were asked if they agreed to have their interviews audio and video recorded and if they would like to be contacted for potential participation in the co-design event. For full details on the methods used, refer to the study by MacPherson [24].

#### Creating the Short Film

Following the interviews, a short film was created featuring interview clips to highlight key “touchpoints” or themes and outline experiences within the patient journey. Only participants who consented to their video being used were included in the study. Following thematic analysis, salient quotes were identified, and the corresponding video clips were spliced into a 10-minute film created by Fraser Health’s Virtual Health department. The film is an essential element of the EBCD approach, which captures patient experiences to help facilitate conversation in stage 4, and is an effective tool to communicate patient experiences to health care providers.

### Stage 4: Co-Design Event

Interview participants who consented to be contacted, steering committee members, and clinicians were invited to an in-person co-design workshop where the touchpoint film from stage 3 was presented to stimulate discussion on improving virtual care. The film highlighted key experiences and themes identified in the interviews, providing a foundation for the co-design discussions. Community members were introduced to the principles of co-design and engaged in collaborative problem-solving activities to address 6 key challenges: language barriers, digital literacy, comfort and convenience, suitability, quality of care, and awareness of available services.

These 6 challenges were derived from thematic analysis of participant interviews conducted in stages 2 and 3, representing the most salient and recurrent themes reported by participants. The steering committee reviewed and confirmed these challenges before the workshop to ensure that they accurately reflected participant priorities. Discussion prompts and structured design thinking exercises were specifically aligned with these areas, ensuring that participant contributions addressed the most relevant issues for improving the virtual hospital-at-home service.

Participants contributed ideas through structured design thinking exercises, including the “5 Whys” technique and “Crazy 8’s” brainstorming method. The “5 Whys” is a root cause analysis method that involves asking “why” iteratively (typically 5 times) to drill down to the underlying cause of a problem [25]. “Crazy 8’s” is a rapid ideation exercise in which participants sketch 8 distinct ideas in 8 minutes, promoting creative thinking and diverse solution generation [26,27]. Both methods facilitated active engagement and helped structure participant feedback. The proposed solutions were analyzed and subsequently shared with the virtual hospital-at-home development team for further consideration.

### Stage 5: Co-Design Analysis, Follow-Up, and Internal Uptake

Although a formal celebration event (stage 6 in the traditional EBCD model) was not conducted, several postworkshop activities were carried out to ensure continuity and uptake of insights. Ideas generated during the co-design event were thematically analyzed by the research team to identify crosscutting recommendations. These recommendations were then shared with the virtual hospital-at-home development team, who reviewed and discussed the feasibility of incorporating them into ongoing service planning.

In addition, follow-up debrief sessions were held with the steering committee to reflect on the process, discuss next steps, and disseminate knowledge products (eg, the touchpoint film and a summary of co-design insights). Informal celebrations occurred during these meetings, where the steering committee members acknowledged the value of the process and its outcomes.

### Ethical Considerations

This project received ethical approval from the Fraser Health Research Ethics Board (FHREB; approval IDs H23-03144 and H23-00463). All participants provided written informed consent prior to participation, including separate consent for video recording, for inclusion of video footage in a short film shared at the co-design event, and for potential follow-up contact regarding subsequent co-design activities. Participants were informed of the purpose and procedures for each stage of the project, as well as their right to withdraw at any time without consequence.

All data were treated confidentially and stored on secure, password-protected servers accessible only to the research team. Identifying information was removed or anonymized in transcripts and reports to protect participant privacy. Video recordings were used solely for analysis and, where consent was provided, for inclusion in the co-design film. The film was shown only to event participants and was not distributed beyond that setting.

No financial or other forms of compensation were provided for participation in the co-design event.

## Results

### Stage 1: Establishing a Steering Committee

A total of 13 individuals participated in the steering committee, representing a range of end-user groups, including representatives from the PICS society; Fraser Health’s South Asian Health Institute; Fraser Health Virtual Health; Fraser Health’s Equity, Diversity, and Inclusion team; and academic researchers from across Canada. Over the course of 12 months, the committee held 8 meetings, with an average attendance rate of 8 members (61% of the committee).

Early discussions focused on refining the research approach, with members advocating for culturally relevant interview questions within stage 3 and the inclusion of language support services to improve accessibility. As data collection progressed, committee members played a critical role in interpreting the findings, offering insights that contextualized patient experiences within the broader health care system.

### Stage 2: Observing Service Delivery and Gathering Staff Experiences

As reported in the study by N Moghaddam, M Montenegro, and M MacPherson (unpublished data, 2025), 7 staff interviews (n=5, 71% physicians and n=2, 29% registered nurses and n=5, 71% women and n=2, 29% men) highlighted both opportunities (extended assessment time and stronger therapeutic relationships) and barriers (limited involvement in early development stages) in remote monitoring. These findings reinforced the importance of engaging clinicians in program design and informed subsequent co-design discussions in this project.

### Stage 3: Gathering Patient Experiences and Creating a Short Film

As reported in the study by MacPherson [24], 20 interviews with South Asian community members identified as patients or caregivers explored attitudes, barriers and facilitators, and suggestions for enhancing awareness and improving virtual care services. While participants were generally open to virtual hospital-at-home models, they identified barriers related to trust, limited awareness, and challenges in access.

From these interviews, a 10-minute touchpoint film was created to highlight the key experiences and themes identified by participants. Of the 20 participants, 9 (45%) consented to inclusion (n=6, 67% women and n=3, 33% men; aged between 19 and 69 y), with 3 (15%) opting for audio-only contributions. Participants included patients (n=3, 33%), caregivers (n=2, 22%), and those who identified as both patients and caregivers (n=4, 44%). Video and audio clips were selected to represent the most salient themes identified in the interviews, such as building trust in virtual care, addressing language barriers, and ensuring equitable access. The film, produced by Fraser Health’s Virtual Health department, served as a central EBCD tool to ground the subsequent co-design event in authentic patient and caregiver voices.

### Stage 4: Co-Design Event

#### Overview

A total of 46 individuals were invited to participate in the co-design workshop, including interview participants from stage 3 who consented to be contacted, steering committee members, and clinicians. Participants with divergent views, including those who were initially skeptical of virtual care, were purposefully included in the co-design session to ensure a broad range of perspectives and stimulate rich discussion. Ultimately, 11 participants from stage 3 attended, alongside 6 steering committee members, a physician, 2 clinical nurse specialists, a learning consultant for Virtual Health, the manager for clinical operations for the VPU, and a coordinator for the South Asian Health Institute.

This workshop aimed to generate solutions to the problem areas identified during the interviews in stages 2 and 3, addressing the five key themes described here: (1) patient-centered and equitable care; (2) technology, innovation, and digital literacy; (3) training and education; (4) community engagement and outreach; and (5) process improvement and data use.

#### Patient-Centered and Equitable Care

While many participants appreciated the structure and direct interactions of hospital-based care, they emphasized the importance of ensuring that virtual hospital-at-home models provide the same level of time, reassurance, and guidance. However, ensuring equitable access to care requires addressing cultural and linguistic barriers, which go beyond interpretation and using plain language to include cultural nuance and understanding. For example, some South Asian families may not trust virtual care as “real” care, especially in serious conditions. They may believe that the patient is being dismissed too early or not taken seriously. Therefore, health care providers may need to explain the concept in culturally resonant terms—perhaps likening it to home visits from trusted family physicians in culturally familiar contexts. Community consultations and continuous feedback mechanisms were highlighted as essential to ensuring that health care services align with patient needs.

Participants suggested that health care models should incorporate family caregivers, including younger family members who may play a significant role in patient support. Furthermore, addressing technical concerns and ensuring that digital health care solutions are user-friendly with 2-way communication channels can alleviate patient anxiety, fostering a seamless integration of innovative care models.

#### Technology, Innovation, and Digital Literacy

Digital literacy emerged as a critical factor influencing virtual health care access and engagement. Participants acknowledged that while artificial intelligence and other digital technologies could enhance health care services, discomfort or unfamiliarity with new technology remained a significant barrier. Many patients expressed the need for hybrid digital literacy training that incorporates both online and in-person instruction, ensuring that those unfamiliar with technology can develop confidence in using digital health tools.

The use of body mapping tools with glossaries of translations, accessible via tablets or other digital devices, was suggested as a way to improve communication for patients experiencing language barriers. Participants also emphasized the need for a 24/7 patient support line to assist with technological issues, ensuring continuous access to health care services, particularly in case of an emergency while at home. Transparency in wait times and care escalation pathways, particularly through online queue systems or digital “call bells” (ie, digital notification systems for patients to request timely assistance remotely, similar to a call bell used in hospital), was seen as a potential strategy for improving patient trust and engagement in digital health care solutions.

#### Training and Education

Participants highlighted the need for enhanced training and education for both health care providers and patients. Digital resources, such as instructional videos, were identified as effective tools for increasing accessibility to health care information. Simulation-based learning was widely recommended, with participants advocating for hands-on training before patient discharge to ensure they understand their care plans and expectations. Training for health care providers should also emphasize virtual health competencies, equipping them with the skills necessary to navigate virtual care and digital platforms effectively, ultimately improving patient care.

Cultural competency training was another key recommendation, ensuring that health care providers can communicate effectively with diverse patient populations. To address health literacy challenges, participants suggested the use of multilingual training manuals with visual aids and simplified language to enhance patient comprehension. Participants also recommended updating patient training modules continuously based on current questions and concerns. Addition, structured patient education that clarifies health care processes was seen as essential for helping patients navigate the complexities of the health care system, with a need for clear, step-by-step education, especially regarding the “entry points” of the health care system.

#### Community Engagement and Outreach

Participants underscored the importance of community-driven approaches to health care engagement and education. Meeting patients where they are, through initiatives such as community pop-ups, outreach in long-term care facilities, and targeted engagement in hospice settings, was identified as a key strategy for increasing health care accessibility. To build motivation to try virtual hospital-at-home models, participants recommended engaging in transparent discussions with patients as to why the virtual hospital-at-home model would be most suitable to their individual circumstances.

Peer learning was also seen as an effective method for knowledge transfer, particularly among older adults who may feel more comfortable learning from their peers. Social media and online communities were highlighted as valuable platforms for sharing health care experiences, testimonials, and educational content. Addition, participants suggested leveraging community champions and patient testimonials to promote new health care models and build trust in virtual health solutions. Media and promotional campaigns, particularly in South Asian media networks, were also recommended to increase public awareness and encourage informed decision-making about health care options.

#### Process Improvement and Data Use

Participants emphasized the role of research, data collection, and continuous feedback in improving health care processes. Both qualitative and quantitative data collection were seen as essential for refining care models and ensuring that they meet patient needs. Global information sharing was recommended as a way to integrate best practices from different health care systems. Participants also stressed the importance of transparency in care escalation processes, ensuring that patients understand when and how to seek additional support. Determining the appropriate fit between hospital-based and virtual care was seen as crucial for optimizing resource allocation and patient outcomes. Additionally, surveys and patient reviews were suggested as mechanisms for evaluating health care experiences and identifying areas for improvement. By bridging the gap between traditional and digital health care models, participants believed that the health care system could become more efficient, accessible, and responsive to patient concerns.

### Stage 5: Post–Co-Design Analysis, Follow-Up, and Internal Uptake

Thematic analysis of workshop data resulted in a set of actionable recommendations, which were shared with the virtual hospital-at-home development team. Internal team discussions helped prioritize feasible ideas for implementation. While a formal evaluation was not conducted, follow-up meetings with the steering committee provided an opportunity to reflect on the success of the project and explore future applications of co-design. Members expressed appreciation for the participatory nature of the process, and the knowledge products were circulated for a broader impact. One participant shared that the co-design event was the first time they felt truly heard by the health care system, and it motivated them to explore a career in health care—a powerful testament to the impact of the process.

## Discussion

### Principal Findings

This study demonstrates the value of using an EBCD approach to inform the development of a patient-centered and equitable virtual hospital-at-home model within a large, culturally diverse regional health authority. By meaningfully engaging patients, caregivers, health care providers, and community organizations across 5 structured stages, the project not only surfaced critical insights into patient experiences but also helped shift traditional power dynamics by positioning patients and caregivers as partners in the design process. This participatory approach generated equity-focused recommendations that highlight how virtual hospital-at-home services can be more responsive to the needs of underserved communities.

Consistent with previous evidence showing that virtual hospital-at-home models improve outcomes and reduce hospital use [2], our findings confirm the promise of this care model. However, we also extend previous research by showing that successful and equitable implementation requires more than technology alone. Participants emphasized that access is contingent on culturally safe, linguistically appropriate, and caregiver-inclusive approaches. For example, as described in the stage 3 interviews, some South Asian families perceived virtual care as less legitimate than hospital-based services, underscoring the importance of culturally resonant communication strategies.

Digital literacy, caregiver inclusion, and culturally competent communication emerged as pivotal enablers of trust and adoption. Participants in the stage 4 co-design event proposed innovative solutions, such as hybrid training that combines online and in-person instruction, the use of body mapping with multilingual glossaries, and caregiver-centered education modules.

Similarly, digital literacy and caregiver involvement emerged as critical enablers of trust and engagement, reflecting themes raised in the stage 4 co-design workshop. Participants’ proposals, such as hybrid digital literacy training, multilingual educational tools, and caregiver-centered education, illustrate how community-driven insights can translate into practical solutions. These grassroots innovations, surfaced through authentic engagement, demonstrate how EBCD operates not only as a design thinking framework but also as a platform for empowerment, amplifying voices that are often marginalized in top-down service planning.

At the same time, perspectives from clinicians and health system staff revealed important implementation challenges. As seen in stage 2 interviews, while health care providers valued the potential of virtual care (eg, extended assessment time and earlier intervention), many reported feeling excluded from initial design processes, which limited their confidence in making referrals. This disconnect reflects a broader challenge in virtual health: aligning patient-centered priorities with operational realities. Previous research highlights that clinician engagement early in design processes is critical to ensuring that virtual care integrates seamlessly into workflows [28]. Our findings reinforce this point, suggesting that virtual hospital-at-home models cannot succeed without parallel attention to patient and health care provider perspectives.

### Implications for Practice and Policy

This study highlights 5 practice and policy implications to support equitable virtual hospital-at-home implementation, grounded in our findings and supported by the existing literature.

#### Culturally Responsive Design

Virtual models must be co-developed with, not simply for, cultural communities. Tailored communication, education, and engagement strategies are necessary to build trust and legitimacy. This aligns with previous work emphasizing that digital health interventions must be culturally adapted to reduce health inequalities [29] and that co-design uncovers culturally resonant strategies that improve uptake [22].

#### Hybrid Digital Literacy Support

Bridging digital divides requires blended approaches that combine in-person training, peer-led instruction, and multilingual resources. As recommended by stage 4 participants, hybrid digital literacy models ensure that those unfamiliar with technology are supported while maintaining flexibility for more experienced users. Evidence suggests that such models enhance confidence and adoption among populations with limited previous exposure to digital tools [30,31].

#### Ongoing Feedback Loops

Embedding continuous feedback mechanisms (eg, community consultations, patient surveys, and user analytics) ensures that services remain responsive to evolving needs. This is consistent with evidence that iterative engagement strengthens program equity and sustainability [32].

#### System Integration and Escalation Clarity

Clear care pathways and escalation protocols are essential for patient safety and trust. In our study, participants requested transparent communication about when and how to escalate concerns, echoing previous research demonstrating that virtual hospital models can be sustainably embedded into broader care systems through shared “digital front doors,” referral pathways, and record sharing that support continuity of care [33].

#### Caregiver Inclusion

Caregivers were consistently identified as central to the success of virtual hospital-at-home models. Equipping caregivers with tools, training, and access to participate directly in care planning not only supports patients but also reduces barriers related to language and technology. Previous studies confirm that caregiver involvement, including proxy access to digital platforms, enhances access and equity for underserved groups [34-36].

#### Broader Policy Recommendations

Beyond these practice implications, broader policy action is needed. Standardizing culturally competent practices, investing in multilingual digital tools, and establishing mechanisms for community governance in digital health initiatives are critical next steps. As frameworks for patient-centered telemedicine suggest, community involvement and cultural adaptation must be embedded as core policy principles rather than optional add-ons [37].

### Limitations

This study has several limitations. First, the interviews focused exclusively on South Asian communities. Although intentional, given the demographic composition of the Fraser Health region, future work should include additional cultural and linguistic groups to ensure broader applicability. Second, the co-design process produced recommendations but did not include a formal evaluation of their implementation or impact. Assessing the outcomes of these interventions will be essential for determining their sustainability and effectiveness. Finally, at the time of data collection, Fraser Health had only launched a single virtual hospital-at-home service, the VPU, and had not yet determined which other clinical populations would be prioritized for future expansion. Consequently, our findings do not speak of the implementation of a specific model beyond psychiatry. However, this early engagement with patients and caregivers was intentional; by eliciting perspectives before clinical parameters were finalized, the co-design process generated insights that can shape a more patient-centered and equitable foundation for service development across diverse populations.

### Reflections and Challenges of Implementing EBCD in a Health System Context

Implementing EBCD in a large and complex health system context such as Fraser Health presented unique challenges and valuable learning opportunities. While the process effectively centered on patient voices and stimulated rich discussions, it also disrupted the status quo. Recruiting physician participants proved difficult, despite outreach efforts; only one physician (an acting Medical Information Officer) attended the co-design session. This highlights the tension between clinical demands and the time investment required for meaningful engagement in participatory design.

In addition, this project was not embedded within the core design cycle of the virtual hospital-at-home program, which limited the extent to which recommendations could be immediately integrated. Some suggestions generated during the workshop also fell outside the direct influence of the Fraser Health team, highlighting the need for stronger system-wide support for patient-driven ideas and cross-organizational collaboration.

Nevertheless, the project demonstrated the value of partnership with community organizations and the importance of cultural and linguistic accessibility in participatory research. Language accommodations during interviews improved accessibility, and the use of a video touchpoint film was particularly effective in centering patient stories and perspectives during the workshop. Divergent views, such as a participant strongly opposing virtual care, proved essential in challenging assumptions and underscoring the diversity of opinions within the patient population. These perspectives enriched the discussion and ultimately strengthened the relevance of the proposed solutions.

A final lesson learned was that for many health system users, engaging in a process without a guaranteed “end product” (such as a finalized service design) was a leap of faith. Although the impact of the workshop was palpable, participants expressed feeling heard, empowered, and connected. For some, it was a transformative experience, reinforcing the need for participatory approaches in health service design that go beyond consultation to foster real collaboration.

### Future Directions

Building on this foundational work, future efforts should prioritize piloting and evaluating co-designed interventions across diverse populations and clinical settings. There is also a need to explore how EBCD can be embedded as a routine part of virtual service development rather than used episodically. As technology continues to evolve, virtual care systems must be supported by ongoing, rather than one-time, engagement processes to remain equitable, culturally relevant, and trusted by the communities that they aim to serve.

### Conclusions

This project illustrates how EBCD can serve as an empowering approach to guide the development of equitable and patient-centered virtual hospital-at-home services. By engaging patients, caregivers, health care providers, and community leaders throughout the co-design process, the project identified both barriers and actionable strategies for improving virtual care delivery. These findings underscore the importance of integrating cultural competence, digital literacy, caregiver inclusion, and systems-level coordination into virtual care models, particularly when serving diverse populations. As virtual hospital-at-home programs continue to expand, intentional and participatory approaches such as EBCD will be essential to ensuring that these innovations are not only effective but also inclusive, sustainable, and trusted by those they are designed to serve. To ensure that virtual care achieves its full promise, future initiatives must embed equity and patient partnership as core, not optional, design principles.

## Abbreviations

EBCD: experience-based co-design
PICS: Progressive Intercultural Community Services
VPU: virtual psychiatric unit
